# 

**Published:** 1890-10-04

**Authors:** 


PRICE ONE PENNY.

No. 210, Vol. IX.—October 4th, 1890.

[Registered at the General Post Office as a Newspaper for transmission in the United Kingdom and Abroad.]

WITH EXTRA NURSING SUPPLEMENT.

Per Annum, 6s. 6d.; post free.

Monthly Parts, 6d.; post free 8d.

Ditto per Annum, 8s., post free.

### THE HOSPITAL

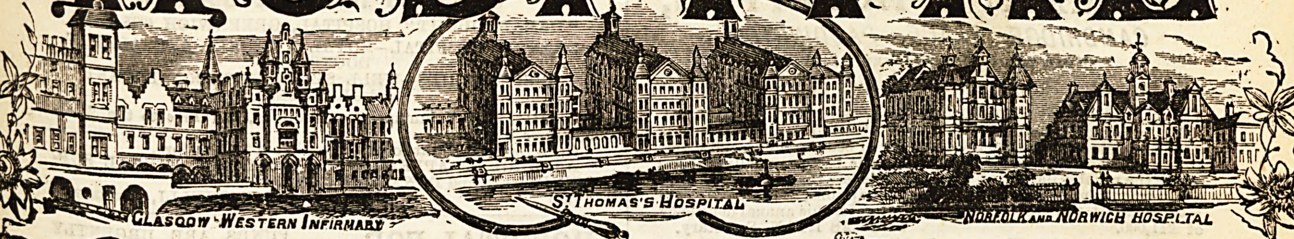

A WEEKLY JOURNAL OF
Science, Medicine, Nursing and Philanthropy.

SATURDAY, OCTOBER 4th, 1890.

**Pugilistic Science** - 1
**Words of Consolation.**—Loneliness - 2
**The Doctor's Pulpit.**—Props and Buttresses - 3
**There and Back** - 4
**Manual Training in Schools.**—I. Its Use and Abuse - 5
**The History and Capabilities of Herbal Simples.** By W. T. Fernie, M.D. XX.—Club Moss - 6
**Everybody's Page** - 7
**Notes and News** - 8
**The Story of the Insane from Year to Year** - 9
**Annotations.**—A Wife to Wives—A Perennial Question—Portobello Philosophers - 10
**The Practitioner's Mirror and Retrospect:—**
MEDICINE—Disease of the Pancreas - 11
SURGERY—Deglutitio Impedita - 12
THERAPEUTICS—A New Invalid Dress - 13
**Death Certificates and Murder** - 14
**Students' Column** - 14
**Editor's Letter-box**—Mental Derangement and Outward Surroundings—"No Gentleman keeps a Bull Dog" - 15
**A Hint for British Artists** - 15
**Passing Topics.**—The C.O.S.'s Protest - 16
**General Charities** - 16
**Scraps and Gleanings** - 16

EXTRA SUPPLEMENT—THE NURSING MIRROR.

**En Passant.**—Nursing in India—The Advance of the Private Nurse—Soldiers' Wives as Nurses—Short Items—Home of Rest at Bognor—In California—Midwife or Charwoman? - i

**The Lifting and Moving of Patients** - ii

**Six Months in a Hospital.** By a "Special Pro."—II. - iii

**Everybody's Opinion.**—Male Nurses - iii

**Lectures** - iv

**Appointments** - iv

**Notes and Queries** - iv

**Vacancies** - iv

**Amusements and Relaxation** - iv

